# Acute kidney injury contributes to worse physical and quality of life outcomes in survivors of critical illness

**DOI:** 10.1186/s12882-022-02749-z

**Published:** 2022-04-07

**Authors:** Kirby P. Mayer, Victor M. Ortiz-Soriano, Alborz Kalantar, Joshua Lambert, Peter E. Morris, Javier A. Neyra

**Affiliations:** 1 Department of Physical Therapy, College of Health Sciences, University of Kentucky, 900 Rose Street, Lexington, KY 40536 USA; 2 Center for Muscle Biology, College of Health Sciences, University of Kentucky, Lexington, KY USA; 3 Kentucky Research Alliance for Lung Disease, College of Medicine, University of Kentucky, Lexington, KY USA; 4 Division of Nephrology, Bone and Mineral Metabolism, University of Kentucky Medical Center, 800 Rose Street, MN668, Lexington, KY 40536 USA; 5 School of Medicine, University of Louisville, Louisville, KY USA; 6 College of Nursing, University of Cincinnati, Cincinnati, OH USA; 7 Division of Pulmonary, Critical Care and Sleep Medicine, Department of Internal Medicine, University of Kentucky, Lexington, KY USA

**Keywords:** Acute kidney injury; critical illness, Patient-centered outcomes, Quality of life, Survivors

## Abstract

**Objectives:**

Survivors of critical illness and acute kidney injury (AKI) are at risk of increased morbidity. The purpose of this study was to compare physical, emotional, and cognitive health in survivors of critical illness with and without AKI.

**Methods:**

Retrospective cohort study of adult (≥ 18 years old) survivors of critical illness due to sepsis and/or acute respiratory failure who attended follow-up in a specialized ICU Recovery Clinic. Outcomes were evaluated during 3-month visit and comprised validated tests for evaluation of physical function, muscle strength, cognitive and emotional health, and self-reported health-related quality of life (HRQOL). Descriptive statistics and group comparisons were performed.

**Results:**

A total of 104 patients with median age of 55 [49-64] years, 54% male, and median SOFA score of 10 [8-12] were analyzed. Incidence of AKI during ICU admission was 61 and 19.2% of patients required renal replacement therapy (RRT). Patients with AKI stage 2 or 3 (vs. those with AKI stage 1 or no AKI) walked less on the 6-min walk test (223 ± 132 vs. 295 ± 153 m, *p* = 0.059) and achieved lower of the predicted walk distance (38% vs. 58%, *p* = 0.041). Similar patterns of worse physical function and more significant muscle weakness were observed in multiple tests, with overall worse metrics in patients that required RRT. Patients with AKI stage 2 or 3 also reported lower HRQOL scores when compared to their counterparts, including less ability to return to work or hobby, or reengage in driving. There were no significant differences in cognitive function or emotional health between groups.

**Conclusions:**

Survivors of critical illness and AKI stage 2 or 3 have increased physical debility and overall lower quality of life, with more impairment in return to work, hobby, and driving when compared to their counterparts without AKI or AKI stage 1 at 3 months post-discharge.

**Supplementary Information:**

The online version contains supplementary material available at 10.1186/s12882-022-02749-z.

## Introduction

Acute kidney injury (AKI) is a common complication occurring in about half of critically ill patients admitted to the Intensive Care Unit (ICU) [1, 2]. Incident AKI is associated with increased morbidity and mortality, prolonged hospitalization, and higher resource utilization, particularly in critically ill patients [3, 4]. Importantly, survivors of hospitalization with AKI are at increased risk of developing kidney disease progression and cardiovascular disease. Traditionally, clinical research in AKI has focused on epidemiology, risk-classification and therapies to prevent AKI and/or post-AKI clinical outcomes, with little focus on patient-centered outcomes, such as physical function and quality of life in survivors of AKI. This is particularly important in the context of AKI during critical illness as survivors of critical illness are known to have high risk of persistent impairments in physical [5, 6], cognitive [7, 8], and emotional health [9, 10], which may be exacerbated in those surviving critical illness complicated with AKI [11].

Survivors of critical illness who suffer physical, cognitive or emotional impairments have difficulties with activities of daily living [12], reduced likelihood of returning to work [13] and driving [14], and overall worse health-related quality of life scores (HRQOL) [15]. The receipt of renal replacement therapy (RRT) has been recognized as a potential risk factor for developing physical impairments [16–18], and emotional health symptoms [19, 20] in ICU survivors. However, incident AKI has not been comprehensively studied as a risk factor for worse patient-centered outcomes, particularly in survivors of critical illness.

The main objective of this study was to determine if patients surviving critical illness complicated with AKI had worse patient-centered outcomes compared to ICU survivors without AKI followed in a dedicated ICU Recovery Clinic after hospital discharge. We hypothesized that survivors of critical illness and AKI have worse physical and cognitive function performance, and overall worse quality of life when compared to ICU survivors without AKI.

## Methods

### Study design and patients

We conducted a retrospective cohort study of survivors of critical illness who were admitted to the ICU at the University of Kentucky Hospital and were followed in a dedicated ICU Recovery Clinic from January 1, 2018 to January 31, 2021. This study was approved and informed consent was waived during medical expedited review by the Institutional Review Board at the University of Kentucky (MEDXP #47751).

Adult ICU patients (≥ 18 years old) surviving acute respiratory failure and/or sepsis received referral to the ICU Recovery Clinic as standard of care. The protocols, timeline, and team of this clinic have been previously described [21]. Briefly, the purpose of this clinic is to provide interdisciplinary care to ICU survivors in the acute critical illness recovery phase with focus on the first 12 months following hospital discharge. In this study, patients who attended the 3-month visit were included. Patients were excluded from the study if they had history of end-stage kidney disease, kidney transplant or if they had a new or pre-existing diagnosis of an acute neurologic or traumatic injury that prevented participation in outcomes testing at the 3-month outpatient visit. AKI was defined according to the Kidney Disease: Improving Global Outcomes (KDIGO) criteria [22]. The receipt and duration of RRT were assessed as well as the dependence on RRT at the 3-month visit to the ICU Recovery Clinic.

### Study outcomes

Patient-centered outcomes were organized in 4 categories: 1) physical function; 2) HRQOL; 3) cognitive function; and 4) emotional health. This battery of tests was implemented as standard of care in the ICU Recovery Clinic as per expert recommendations for survivors of acute respiratory failure [23].

#### Physical function and muscle strength

1) The distance a patient ambulated on the six-minute walk test (6MWD), in which longer distance indicates better exercise capacity and physical function [6]; 6MWD was assessed as the absolute distance in meters and the percentage achieved of the predicted distance [24]; 2) Short Physical Performance Battery (SPPB), a measure of lower-extremity physical function including components of standing balance, habitual 4-m gait speed, and chair stand test; higher scores on SPPB indicate better physical function [25, 26]; and 3) Medical Research Council-sum score (MRC-ss) to assess global peripheral muscle strength in 12 defined muscles (scores range from 0 to 60; lower scores indicate muscle weakness).

#### HRQOL

1) Euro-Quality of Life (EQ-5D), which is self-reported HRQOL on a visual analog scale (VAS) ranging from 0 to 100; higher scores indicate better quality of life [27, 28]; 2) Self-reported return to work (for patients previously employed) or to hobby (for retired individuals); and 3) Self-reported return to driving (if individuals used to drive prior to hospitalization).

#### Cognitive function

Montreal Cognitive Assessment (MOCA), which is a test to assess cognitive function with scores < 23/30 identifying mild cognitive impairment [29, 30].

#### Emotional Health

1) Hospital Anxiety and Depression Scale (HADS), a fourteen item questionnaire containing components of anxiety and depression [31]; and 2) Impact of Events Scale-Revised (IES-R), a twenty two-question self-reported questionnaire assessing emotional distress; a provisional diagnosis of post-traumatic stress disorder could be determined based on a cut-off score of > 33/88 [32].

### Clinical data and definitions

Demographic variables of age, sex, race/ethnicity, and body mass index (BMI) were extracted from the electronic health record (EHR). Charlson score was calculated to assess comorbidity burden using ICD-10 codes from the index ICU admission. Clinical data from the EHR included sequential organ failure assessment (SOFA) [33] assessed within the first 48 h of ICU admission, sedative and agitation status as measured by the mean score on the Richmond Agitation Sedation Scale (RASS) [34] in the first 72 h of ICU admission, receipt and duration mechanical ventilation, receipt of tracheostomy, receipt of extracorporeal membrane oxygenation (ECMO), receipt and frequency of physical and occupational therapy during admission, receipt of vasopressor or inotropes, receipt of steroids, receipt of neuromuscular blockers (NMB), ICU length of stay (LOS), hospital LOS, and discharge destination. Clinical data from the ICU Recovery Clinic included all-cause rehospitalization or emergency department utilization since discharge from the index hospitalization up to 90 days of follow-up. Laboratory values including creatinine (highest) and hematocrit (lowest) were extracted at three time points 1) within first 48 h of ICU admission, 2) hospital discharge and 3) time of ICU Recovery Clinic follow-up (+/− 30 days). Anemia was defined as hematocrit < 39% for males and < 36% for females [35] and estimated glomerular filtration rate (eGFR) were calculated by CKD-EPI equation [36].

### Statistical analysis

Descriptive statistics and histogram visualization were performed to assess the central tendency (mean or median) and the variations in the data (stand deviation [SD] or interquartile range [IQR]). Normality was assessed with Shapiro-Wilk test and the appropriate parametric and non-parametric tests for comparisons were employed. For two-group comparisons, independent t-test or Mann Whitney U-test were used for continuous variables and Chi-square or Fisher’s exact test for categorical variables. We compared patient characteristics and outcomes between two groups: 1) patients without AKI or mild AKI (stage 1 AKI) and 2) patients with severe AKI (stage 2 or 3). For three-group comparisons, one-way ANOVA and chi-square tests were utilized as appropriate. These groups included: 1) patients without AKI or AKI stage 1; 2) patients with AKI stage 2 or 3 that did not receive RRT; and 3) patients with AKI requiring RRT during the index admission. Exploratory multivariable logistic and linear regression models were constructed to investigate the relationship between incident AKI (independent variable) and selected study outcomes. All models were adjusted by sex and index SOFA and Charlson scores. Additional candidate variables (age, BMI, days of mechanical ventilation, receipt of NMB, receipt of vasopressor/inotrope, receipt of ECMO, and hospital LOS) were included into the regression model if their multivariable *p*-value was ≤0.05. Significance was determined with an alpha level set at *p* ≤ 0.05 (2-tailed). R version 4.1.0 and Prism 9.3 (GraphPad, San Diego, CA) were used for statistical analysis.

## Results

### Patient characteristics

**Fig. 1:**
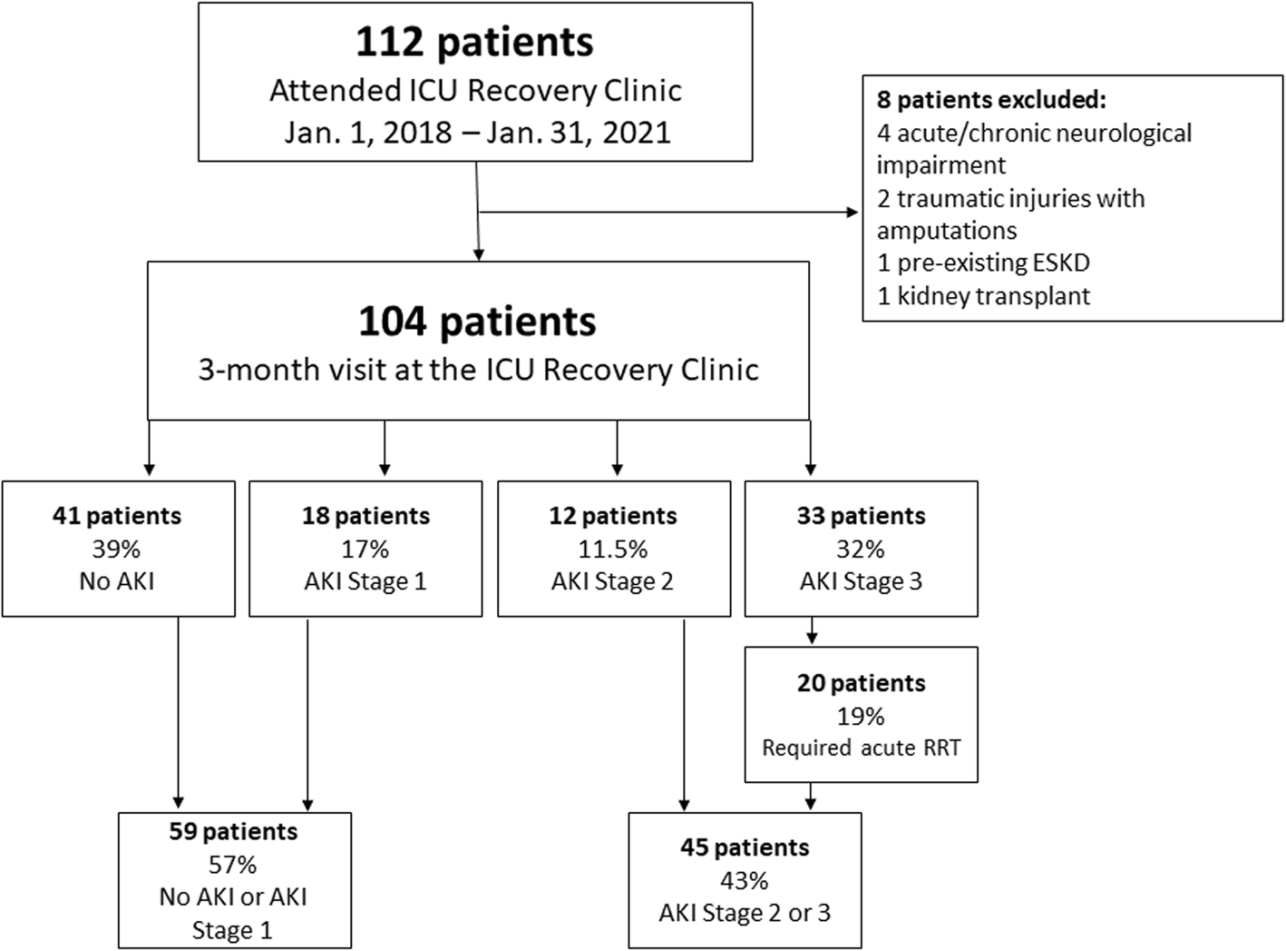
Consort diagram with study patients stratified by AKI status

A total of 112 patients attended appointment at the ICU Recovery Clinic during the study period. Among these, 104 patients met study criteria (Fig. 1). Patients included in the study had a median (IQR) age of 55 [49 – 64] years-old, 54% were male, 27% were of a non-white race/ethnicity, and their median (IQR) index SOFA score was 10 [8 – 12]. Forty-one (39%) of ICU survivors did not have AKI during their admission. Among the 63 (61%) patients who suffered from AKI, 18 (28.6%), 12 (19%), and 33 (52.4%) had stage 1, stage 2, and stage 3 AKI, respectively. A total of 20 patients required acute RRT during their index hospitalization but none of them were dependent on RRT by the time of the 3-month evaluation in the ICU Recovery Clinic (Fig. 1).

Fifty-nine patients were grouped in the no AKI or AKI stage 1 group (*n* = 59/104; 57%) and 45 patients had AKI stage 2 or greater (*n* = 45/104; 43%; Fig. 1). There were no differences in age, sex, BMI, race/ethnicity or Charlson scores between the two groups. Patients with AKI stage 2 or 3 had longer ICU and hospital LOS, higher index SOFA scores, required more mechanical ventilation with longer durations, received more NMB, had lower hematocrit, met criteria for anemia more frequently, and more frequently underwent tracheostomy when compared to patients without AKI or AKI stage 1 (Table 1). When further stratifying incident AKI based on RRT exposure (3 groups), there were no differences in age, sex, BMI, or Charlson scores. Patients that required acute RRT had higher SOFA scores, longer duration of mechanical ventilation, and longer LOS in the ICU and hospital when compared to their counterparts without exposure to RRT (Supplemental Table 1). Patients requiring RRT (*n* = 20) had a median duration of utilization lasting 8.2 days [IQR 6 – 11.2].

**Table 1:** Clinical characteristics of survivors of critical illness stratified by AKI status during index ICU stay

Parameter	No AKI or AKI stage 1 ***n*** = 59	AKI stage 2 or 3 ***n*** = 45	***P***-value
Age, years, median (IQR)	56 (48 – 64)	53.5 (45 – 62)	*p* = 0.60
Sex, male, n (%)	28 (47)	28 (45)	*p* = 0.17
Race / ethnicity			
White	42 (71)	34 (76)	
Black	9 (15)	10 (22)	*p* = 0.19
Hispanic / Latino	6 (10)	1 (2)	
Other	2 (3)	0	
BMI, kg/m^2^, median (IQR)	35 (29 – 47)	32 (27 – 40)	*p* = 0.49
Charlson score, median (IQR)	2 (1 – 4)	2 (1 – 4)	*p* = 0.73
SOFA score, median (IQR)	9 (5-11)	12 (10 -14)	*p* < 0.001
Mechanical ventilation, n (%)	43 (73)	45 (100)	*p* < 0.001
Days on mechanical ventilation, median (IQR)	8 (5 – 14)	14 (8 – 20)	*p* = 0.009
Tracheostomy, n (%)	10 (17)	16 (36)	*p* = 0.037
ECMO, n (%)	4 (7)	6 (13)	*p* = 0.261
Exposure to steroids, n (%)	33 (56)	30 (67)	*p* = 0.338
Exposure to vasopressors or inotropes, n (%)	35 (59)	33 (73)	*p* = 0.188
Exposure to NMB, n (%)	19 (32)	27 (60)	*p* = 0.004
Sedative status based on RASS, mean ± SD	−1.9 ± 1.6	−3.3 ± 1.3	*p* < 0.001
Serum Creatinine, mg/dl, mean (SD)			
ICU admission (first 48 h)	0.83 ± 0.2	0.87 ± 0.3	*p* = 0.52
Hospital Discharge	0.79 ± 0.3	1.48 ± 1.7	*p* = 0.001
ICU Recovery Clinic follow-up^a^	1.0 ± 0.5	1.33 ± 1.4	0 = 0.23
eGFR, mL/min/1.73 m^2^, mean (SD)			
ICU admission (first 48 h)	101 ± 23	100 ± 27	*p* = 0.89
Hospital Discharge	97 ± 25	72 ± 39	*p* = 0.001
ICU Recovery Clinic follow-up^a^	77 ± 24	67 ± 29	*p* = 0.18
Hematocrit, %, mean (SD)			
ICU admission (first 48 h)	38.1 ± 5.1	35.7 ± 8.0	*p* = 0.021
Hospital Discharge	35.3 ± 5.6	30.3 ± 6.9	*p* < 0.001
ICU Recovery Clinic follow-up^a^	40.4 ± 5.8	36.1 ± 8.4	*P* = 0.007
Anemia (yes), %			
ICU admission (first 48 h)	41%	64%	*p* = 0.016
Hospital Discharge	64%	96%	*p* < 0.001
ICU Recovery Clinic follow-up^a^	22%	56%	*p* = 0.013
Hospital Rehabilitation			
Physical therapy, yes, n (%)	51 (86)	45 (100)	*p* = 0.010
Time to PT, days, median (IQR)	9 (6 – 13)	13 (7 – 18)	*p* = 0.104
Number of PT sessions, median (IQR)	3 (2 – 4)	4 (3 – 8)	*p* = 0.002
Occupational therapy, yes, n (%)	48 (51)	44 (98)	*p* = 0.009
Time to OT, days, median (IQR)	10 (6 – 14)	13 (9 – 19)	*p* = 0.061
Number of OT sessions, median (IQR)	2 (1 – 4)	4 (2 – 6)	*p* = 0.013
ICU LOS, days, median (IQR)	10 (6 – 15)	18 (11 – 24)	*p* < 0.001
Hospital LOS, days, median (IQR)	15 (11 – 22)	29 (17 – 37)	*p* < 0.001
Discharge Destination, n (%)			
LTAC	5 (8)	5 (11)	
Acute or subacute facility	17 (29)	17 (38)	*p* = 0.453
Home with home health services	17 (29)	14 (31)	
Home without services	20 (34)	9 (20)	

*AKI* acute kidney injury, *BMI* body mass index, *SOFA* sequential organ failure assessment, *NMB* neuromuscular blocker, *ICU* intensive care unit, *eGFR* estimated glomerular filtration rate, *LTAC* long-term acute care facility, *LOS* length of stay, *RASS* Richmond Agitation Sedation Scale

^a^ Data presented for patients with follow-up laboratory testing in clinic; *n* = 27 for no AKI or AKI stage 1; *n* = 23 for AKI stage 2 or 3

### Patient-centered outcomes

#### Physical function and muscle strength

**Fig. 2:**
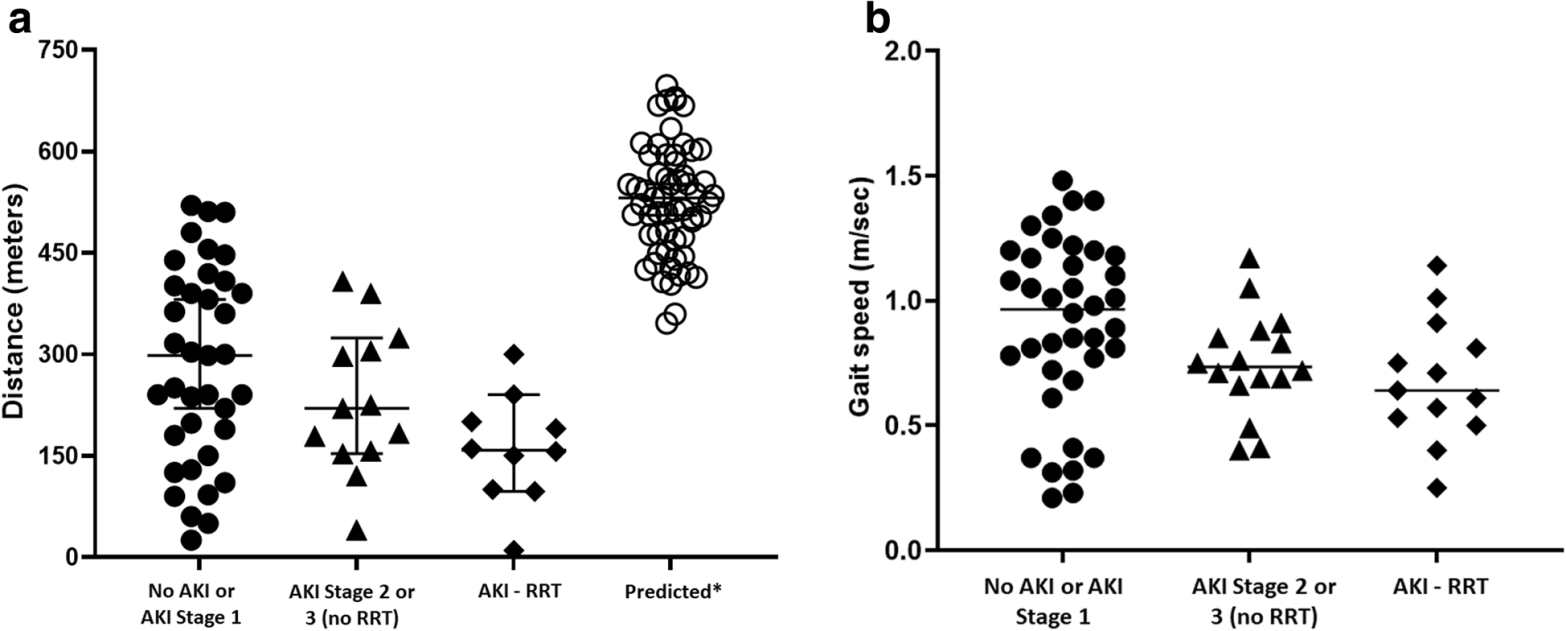
Physical functional assessed 3-months after hospital discharge in survivors of critical illness grouped by severity of acute kidney injury during index ICU admission. Group A = patients with AKI stage 1 or no AKI; Group B = patients with AKI stage 2 or 3 that did not require RRT; Group C = patients with AKI stage 3 that required acute RRT. *Panel A:* Patients that required RRT in the ICU ambulated the shortest distances during six-minute walk test, but this was not statistically different between the three groups (*p* = 0.113). Predicted* represents the distance patients were estimated to achieve based on age, sex, and height. Patients with AKI stage 2 or 3 achieved 38% [IQR: 29 – 56] of their predicted distances compared to 58% [IQR: 34 – 78] in patients with AKI stage 1 or no AKI (*p* = 0.041). *Panel B:* Patients with AKI stage 2 or 3, and especially those that required acute RRT in the ICU had slower 4-m habitual gait speeds when compared to patients with AKI stage 1 or no AKI (*p for 3-group comparison* = 0.031). AKI = acute kidney injury; RRT = renal replacement therapy

Survivors of critical illness that suffered from AKI stage 2 or 3 performed worse on the 6MWD test (i.e. walked shorter distances) when compared to patients with AKI stage 1 or no AKI: 223 ± 132 vs. 295 ± 153 m, *p* = 0.059, respectively. Similarly, survivors of critical illness and AKI stage 2 or 3 achieved less of the predicted walk distance on the 6MWD test than those with AKI stage 1 or no AKI: 38%, IQR: 29-56% vs. 58%, IQR: 34-78%, *p* = 0.041, respectively. (Table 2). In addition, patients with AKI stage 2 or 3 had slower gait speeds than those with AKI stage 1 or no AKI: 0.71 ± 0.22 vs. 0.91 ± 0.3 m/sec, *p* = 0.009, respectively. Patients that required RRT during the index ICU admission achieved the shortest distance on the 6MWD test, the lowest percentage of the predicted walk distance, and the slowest gait speeds when compared to their counterparts without RRT requirement (Fig. 2 and Supplemental Table 2).

**Table 2:** Study outcomes of survivors of critical illness at 3 months following hospital discharge according to AKI status during index ICU admission

Parameter	No AKI/AKI stage 1 ***n*** = 59	AKI stage 2 or 3 ***n*** = 45	***P***-value	Attrition
**Physical function assessment**				
6 MWD, meters, mean ± SD (n)	295 ± 153 (38)	223 ± 132 (25)	*p* = 0.059	39%
6 MWD achieved from predicted, %,median [IQR] (n)	58 [34 – 78] (38)	38 [29 – 56] (25)	*p* = 0.041	39%
Chair Stand Test, sec, median [IQR]	10 [8-13] (38)	12 [10 – 15] (29)	*p* = 0.197	35%
4-m gait speed, m/sec, mean ± SD (n)	0.91 ± 0.3 (38)	0.71 ± 0.22 (29)	*p* = 0.009	35%
SPPB, 0-12, median [IQR] (n)	11 [7 – 12] (38)	8 [5 – 11] (29)	*p* = 0.056	35%
MRC-ss, 0-60, median [IQR] (n)	58 [54 – 60] (38)	54 [48 – 58] (29)	*p* = 0.009	35%
**HRQOL assessment**				
EQ-5D VAS, 0-100, mean ± SD (n)	77.9 ± 14.2 (54)	69.2 ± 20.9 (38)	*p* = 0.019	12%
Return to work/hobby, yes, % (n)	50% (29/58)	22% (10/45)	*p* = 0.003	0%
Return to driving, yes, % (n)	70% (39/56)	46% (19/41)	*p* = 0.016	0%
**Cognitive function assessment**				
MOCA, 0-30, median (IQR)	24.8 ± 3.7 (48)	24.8 ± 3.8 (37)	*p* = 0.861	18%
**Emotional function assessment**				
Anxiety (HADS), 0-21, median [IQR]	6 [2 – 11] (52)	7 [3 – 12] (41)	*p* = 0.270	10%
Depression (HADS), 0-21, mean ± SD (n)	5.3 ± 4.0 (52)	7.0 ± 5.8 (41)	*p* = 0.108	10%
IESR-S, 0-88, mean ± SD (n)	24.7 ± 21.6 (52)	28.8 ± 24.5 (41)	*p* = 0.400	10%
**Clinical assessment**				
90-day hospitalization/ED visit, % (n)	19% (11/59)	29% (13/45)	*p* = 0.247	0%

*HADS* hospital anxiety and depression scale, *IES-R* Impact of Events Scale-Revised, *MOCA* Montreal Cognitive Assessment, *ED* emergency department, *EQ-5D* Euro-Quality of Life- Five-Dimension, *VAS* visual analog scale, *SPPB* short physical performance battery, *MRC-ss* Medical research council-sum scale, *6 MWD* six-minute walk distance

Patients with AKI stage 2 or 3 also exhibited more significant muscle weakness and worse physical function as measured by MRC-ss and SPPB when compared to patients with AKI stage 1 or no AKI (Table 2). A similar pattern of worse outcomes was observed in patients that required RRT vs. those without RRT requirement (Supplemental Table 2). Of the subcomponents of the SPPB, sit-to-stand performance test was not different between groups.

#### HRQOL

Survivors of critical illness with AKI stage 2 or 3 reported lower HRQOL scores on EQ-5D VAS when compared to patients with AKI stage 1 or not AKI: 69.2 ± 20.9 vs. 77.9 ± 14.2, *p* = 0.019, respectively. When stratified into three groups, patients that required RRT had lower HRQOL scores than patients with AKI stage 1 or no AKI, but scores were similar to those of patients that suffered from AKI stage 2 or 3 without RRT (Supplemental Table 2). Patients with AKI stage 2 or 3 were less likely to return to work/hobby and driving compared to patients with AKI stage 1 or no AKI (Table 2). The return to these activities was significantly less achieved in patients that required RRT (Supplemental Table 2).

#### Cognitive and emotional health

Self-reported anxiety, depression, and distress, as well as cognitive function evaluation were not significantly different between groups (Table 2 and Supplemental Table 2).

### Multivariable analysis

In univariable analysis, the occurrence of AKI stage 2 or 3 in reference to AKI stage 1 or no AKI was associated with less distance achieved in the 6MWD test, lower EQ-5D scores, and less chances of returning to work or hobby at 3 months following hospital discharge. After multivariable adjustments, all trends of associations persisted but with attenuated statistical significance (Table 3).

**Table 3:** Multivariable models examining selected outcomes according to AKI status during index ICU admission as the independent variable

Outcome	Model 1	Model 2		Model 3		
**6MWD (meters)**^**a**^						
AKI stage 2-3 vs. ref	−35.9 (−73.3, 1.38)	*p* = 0.059	−21.0 (−62.4, 20.4)	*p* = 0.314	−27.8 (−68.9, 13.3)	*p* = 0.181
**EQ-5D VAS (0-100)**^**a**^						
AKI stage 2-3 vs. ref	−4.35 (−7.98, −0.72)	*p* = 0.020	−4.01 (−8.25, 0.22)	*p* = 0.063	−4.01 (−8.25, 0.22)	*p* = 0.063
**Return to driving (yes/no)**^**b**^						
AKI stage 2-3 vs. ref	0.38 (0.16, 0.87)	*p* = 0.427	0.65 (0.23, 1.87)	*p* = 0.427	0.71 (0.24, 2.10)	*p* = 0.541
**Return to work or hobby (yes/no)**^**b**^						
AKI stage 2-3 vs. ref	0.29 (0.12, 0.68)	*p* = 0.005	0.37 (0.13, 1.05)	*p* = 0.062	0.37 (0.13, 1.05)	*p* = 0.062

Reference (ref) for all models was no AKI or AKI stage 1

Model 1: univariable model

Model 2: adjusted by sex, SOFA score, and Charlson score

Model 3: adjusted by model 2 + candidate variables age, BMI, days of mechanical ventilation, receipt of NMB, receipt of vasopressor/inotrope, hospital LOS, receipt of ECMO according to multivariable *p*-value ≤0.05. Final models included the addition of receipt of NMB (model of 6MWD) and days of mechanical ventilation (model of return to driving)

^a^ Data are presented as beta coefficient and 95% confidence intervals

^b^ Data are presented as odds ratios and 95% confidence intervals

## Discussion

The findings of this study demonstrate that survivors of critical illness and severe AKI (KDIGO stage 2 or 3) have increased risk of physical disability and worse quality of life compared to ICU survivors without AKI or with milder forms of AKI (KDIGO stage 1). A trend towards worse performance on physical function, muscle strength, and exercise capacity was also observed in survivors of critical illness that received acute RRT for AKI vs. those who did not. To our knowledge this is the first study examining patient-centered outcomes 3 months following hospital discharge in survivors of critical illness according to incident AKI during the index ICU admission. The findings of this study are clinically relevant because collectively the data suggest that survivors of critical illness and AKI may have increased risk of physical disability and impaired HRQOL, and thus potentially benefit from therapeutic interventions. Our findings support that survivors of critical illness and AKI, particularly those with AKI stage 2 or 3, should, at minimum, be assessed for physical impairments after hospital discharge.

Survivors of critical illness and AKI that required RRT only achieved a mean distance of 192 m (95% CI: 111-274) on the six-minute walk test performed 3 months post-discharge. This is substantially lower than what was reported in a meta-analysis of survivors of critical illness at the same time-point of evaluation, 361 m (95% CI: 321-401) [37]. Reductions in physical function lead to difficulty with activities of daily living, returning to work, and returning to driving, which considerably impact HRQOL as demonstrated in our study. Interestingly, emotional health and cognitive function were not significantly different according to AKI status in survivors of critical illness represented in this study. One should note that critical illness survivors in this study had high scores of anxiety, depression and distress measures as well as impairments in cognitive function consistent with current literature [8–10]. We suspect that the nature of this high-risk population i.e. patients referred to the ICU Recovery Clinic at our institution represents a heterogeneous group of patients that survived critical illnesses as previously defined [21]; therefore, the comparison of study outcomes according to hospitalized AKI may have precluded adjustment by kidney/critical illness recovery trajectories. The severity of illness and the duration of the illness may be more important predictors of emotional health deficits than the development AKI [21]. A previous study revealed that patients surviving ICU that required acute RRT also did not have differences in emotional health when compared to matched survivors not requiring RRT [20]. Thus, emotional and cognitive health outcomes may not be different simply due to the traumatic experience of critical illness for all ICU survivors independent of the occurrence of AKI and/or need of RRT. Of note, the majority of patients included in our cohort required mechanical ventilation via endotracheal tube and more than half received sedatives, thus, both groups are at risk of emotional and cognitive deficits [38].

Historically, the focus for patients with and surviving AKI and those requiring acute RRT has been on short and long-term morbidity and mortality [2, 3, 39, 40]. These clinical outcomes are certainly important but may not adequately capture the arduous recovery and impairments faced by survivors following discharge. In recent years, clinicians, researchers, patients and caregivers have emphasized the need to assess patient-centric outcomes [41–43]. Recently, AKI has been recognized as a critical risk factor that could affect HRQOL [44, 45]. Specifically, patients with AKI that required RRT in the ICU were shown to have lower scores in physical and mental components of HRQOL when compared with the general population [44]. In a different study, Abdel-Kader et al. demonstrated that AKI survivors were at higher risk of frailty up to 12 months post-discharge when compared to patients without AKI [46]. One should note that frailty in this study was quantified by using an index scale based on clinical judgement [47], without incorporating physical performance metrics as in our study.

We observed that survivors of critical illness and AKI that required RRT had the worst performance on physical function and exercise capacity. It is possible that the receipt of RRT may have physiologic impacts on physical performance through the non-selective removal of amino acids and occurrence of hypophosphatemia which could may lead to negative impact on skeletal muscle function. Our team previously demonstrated that incident hypophosphatemia during continuous RRT was associated with increased requirement of mechanical ventilation, which may be explained by impaired respiratory muscle function [48]. We may further hypothesize that regardless of RRT, the clinical consequences of AKI may also lead to skeletal muscle damage. Previous research has shown a strong relationship between muscle wasting and AKI which may be attributed to altered glucose and protein metabolism as well as oxidative stress [49]. We were unable to assess cellular mechanisms of muscle and physical dysfunction given the observational nature of our study, but our findings may serve to generate hypotheses for future experimental research and clinical trials.

Survivors of critical illness and AKI are also at risk of impariments and symptoms related to post-intensive care syndrome (PICS). In this context, ICU Recovery Clinics have emerged as one approach to assess and treat impairments following critical illness. Severe AKI or the need of acute RRT during index ICU stay should be part of risk-classification tools to identify high-risk patients eligible to receive referral for ICU-after care. Clinically, it is important that performance-based testing, self-report, and index tools are incorporated into post-ICU clinic assessments to fully evaluate deficits, improve prognostication, and guide plan of care development. Interdisciplinary collaborations in post-ICU clinics can further optimize the assessment and treatment of symptoms and impairments related to PICS [14, 50]. Improving technologies such as remote activity tracking (actigraphy) and the emergence of telehealth provide new avenues to assess, educate and deliver rehabilitation interventions to high-risk critical illness/AKI survivors. Telehealth may especially impact equity in post ICU care by reducing financial, transportation, and societal barriers [51].

The study is not without limitations. First, the design of the study may lead to selection bias as only ICU survivors who were ambulatory and able to attend the ICU Recovery Clinic were considered for inclusion in the study. Second, representation bias of the available clinical data at the ICU Recovery Clinic may impact the results. For example, patients that did not have a clinical indication for laboratory data at the 3-month follow-up did not have clinical data available. The clinical trajectories during recovery of critical illness may be an important confounder directly influencing results i.e. patients with low hematocrit at time of testing may perform worse regardless of AKI status at hospitalization. However, clinically we expect that patients will have different trajectories of critical illness recovery based on a multitude of factors such as the index severity of illness, AKI severity, age, co-morbidities and so on, which are clinical parameters that were used in our multivariable models. Third, the sample size of the cohort limited power and further stratification in the examination of outcomes into subgroups. Fourth, this study should be considered as hypothesis-generating given that survivors of critical illness and AKI tended to be overall more acutely ill than their counterparts without AKI and therefore the causal relationship between AKI and patient-centered outcomes could not be ascertained. We attempted to account for this by multivariable analysis but residual confounding of unmeasured variables is still possible. Finally, performance metrics evaluated in this study were all obtained under routine clinical care by trained specialists but aggregated missing data ranged from 0 to 39% given clinicians may have opted to omit certain tests, time restrictions may have precluded testing, or patients may have refused some of them.

Our study has also notable strengths. First, this is the first study evaluating a comprehensive battery of patient-centered outcomes in survivors of critical illness according to a well-defined AKI status during the index ICU admission. Second, all patient-centered outcomes were obtained by trained personnel in a specialized ICU Recovery Clinic. Third, we examined well validated metrics for evaluation of physical function, HRQOL and cognitive and emotional function that are supported by critical care literature. Finally, this study could help with the design and power analysis of clinical trials targeting rehabilitation interventions aiming to improve the well-being of survivors of both critical illness and AKI.

In conclusion, when evaluated at 3 months following hospital discharge survivors of critical illness and severe AKI (KDIGO stage 2 or 3) exhibited more physical debility, lower quality of life, and reduced rates of return to work and driving when compared to survivors of critical illness with mild AKI (KDIGO stage 1) or no AKI during the index ICU admission. The receipt of RRT seemed to further adversely influence these outcomes. These findings emphasize the need to assess patient-centered outcomes in survivors of critical illness and AKI, and more importantly develop effective rehabilitation interventions that could favorably impact the well-being of this debilitated and growing population.

## Supplementary Information

**Additional file 1: Supplemental Table 1**. Clinical characteristics of critical illness survivors stratified by AKI status with or without RRT during index ICU stay.

**Additional file 2: Supplemental Table 2**. Study outcomes of survivors of critical illness at 3 months following hospital discharge according to AKI and RRT status during index ICU admission.

## Data Availability

The authors confirm that the data supporting the findings of this study are available within the article and its supplementary materials. Raw data that support the findings of this study are available from the corresponding author, KPM and JAN, upon reasonable request.
