# Supplementary material for: Acute kidney injury contributes to worse physical and quality of life outcomes in survivors of critical illness
Source: BMC Nephrol. 2022 Apr 7;23:137. doi: 10.1186/s12882-022-02749-z (PMC8991933; doi:10.1186/s12882-022-02749-z)
Supplement: Supplementary file 1 — Additional file 1: Supplemental Table 1. Clinical characteristics of critical illness survivors stratified by AKI status with or without RRT during index ICU stay. [file 12882_2022_2749_MOESM1_ESM.docx]

| **Parameter** | **No AKI or AKI stage 1**  **n = 59** | **AKI stage 2 or 3**  **(no RRT)**  **n = 25** | **AKI-RRT**  **n = 20^[[1]](#footnote-1)^** | **P-value** |
| --- | --- | --- | --- | --- |
| Age, years, median [IQR] | 56 [49 – 64] | 54 [49 – 65] | 52 [48 – 65] | 0.50 |
| Sex, male, n (%) | 28 (47) | 16 (64) | 12 (60) | 0.39 |
| BMI, kg/m^2^, mean ± SD | 34.5 ± 7.0 | 32.4 ± 9.1 | 36.5 ± 9.8 | 0.26 |
| Charlson score, median [IQR] | 2 [1 – 4] | 3 [1 – 5] | 2 [1 – 3] | 0.81 |
| SOFA score, mean ± SD | 8.4 ± 3.5 | 11.2 ± 2.2 | 12.8 ± 2.2 | < 0.001 |
| Mechanical ventilation, n (%) | 43 (73) | 25 (100) | 20 (100) | 0.001 |
| Days of mechanical ventilation, median [IQR] | 8 [5 – 14] | 11 [6 -17] | 17 [11 – 22] | 0.003 |
| Tracheostomy, n (%) | 10 (17) | 5 (20) | 11 (55) | 0.002 |
| ECMO, n (%) | 4 (7) | 2 (8) | 4 (20) | 0.21 |
| Exposure to steroids, n (%) | 33 (56) | 16 (64) | 14 (70) | 0.53 |
| Exposure to vasopressors or inotropes, n (%) | 35 (59) | 14 (56) | 19 (95) | 0.006 |
| Exposure to NMB, n (%) | 19 (32) | 12 (48) | 15 (75) | 0.003 |
| Sedative status based on RASS, mean ± SD | -1.9 ± 1.6 | -2.9 ± 1.4 | -3.7 ± 1.0 | < 0.001 |
| Serum Creatinine, mL/min/1.73 m^2^, mean (SD)  ICU admission (first 48 hours)  Hospital Discharge  ICU Recovery Clinic follow-up^i^ | 0.83 ± 0.2  0.79 ± 0.3  1.0 ± 0.5 | 0.78 ± 0.25  0.94 ± 0.54  1.0 ± 0.25 | 0.99 ± 0.34  2.6 ± 2.4  1.2 ± 1.1 | p = 0.033  p = 0.001  p = 0.125 |
| eGFR, mg/dL, mean (SD)  ICU admission (first 48 hours)  Hospital Discharge  ICU Recovery Clinic follow-up^i^ | 101 ± 23  97 ± 25  77 ± 24 | 106 ± 25  88 ± 29  72 ± 22 | 91.7 ± 29  53.3 ± 42  63.2 ± 33 | p = 0.192  p = 0.001  p = 0.289 |
| Hematocrit, %, mean (SD)  ICU admission (first 48 hours)  Hospital Discharge  ICU Recovery Clinic follow-up^i^ | 38.1 ± 5.1  35.3 ± 5.6  40.4 ± 5.8 | 35.9 ± 6.1  31.0 ± 4.5  37.8 ± 5.4 | 34.5 ± 8.1  26.4 ± 2.5  34.4 ± 4.8 | p = 0.041  p < 0.001  p = 0.009 |
| Anemia (yes), %  ICU admission (first 48 hours)  Hospital Discharge  ICU Recovery Clinic follow-up^[[2]](#footnote-2)^ | 41%  64%  22% | 64%  92%  45% | 65%  100%  67% | p = 0.056  p = 0.001  p = 0.026 |
| ICU LOS, days, median [IQR] | 10 [6 – 15] | 15 [10 – 19] | 22 [17 – 30] | < 0.001 |
| Hospital LOS, days, median [IQR] | 15 [11 – 22] | 21 [14 – 18] | 37 (32 – 48.5) | < 0.001 |

**Supplemental Table 1**: Clinical characteristics of critical illness survivors stratified by AKI status with or without RRT during index ICU stay

AKI = acute kidney injury; BMI = body mass index; SOFA = sequential organ failure assessment; ECMO = extracorporeal membrane oxygenation; NMB = neuromuscular blocker; ICU = intensive care unit; LOS = length of stay; RASS = Richmond Agitation Sedation Scale

1. The median duration of RRT for 20 patients was 8.2 [6 -11.2] [↑](#footnote-ref-1)
2. Data calculated for patients with follow-up laboratory testing n = 27 for no AKI or AKI stage 1; n = 23 for AKI stage 2 or 3 [↑](#footnote-ref-2)
