# Supplementary material for: Acute kidney injury contributes to worse physical and quality of life outcomes in survivors of critical illness
Source: BMC Nephrol. 2022 Apr 7;23:137. doi: 10.1186/s12882-022-02749-z (PMC8991933; doi:10.1186/s12882-022-02749-z)
Supplement: Supplementary file 2 — Additional file 2: Supplemental Table 2. Study outcomes of survivors of critical illness at 3 months following hospital discharge according to AKI and RRT status during index ICU admission. [file 12882_2022_2749_MOESM2_ESM.docx]

| **Parameter** | **No AKI or AKI stage 1**  **n = 59** | **AKI stage 2 or 3 (no RRT)**  **n = 25** | **AKI-RRT**  **n = 20** | **P-value** |
| --- | --- | --- | --- | --- |
| **Physical function assessment** | | | | |
| 6 MWD, meters, mean ± SD (n) | 295.2 ± 152.6 (38) | 247.6 ± 132.9 (14) | 192.4 ± 131.1 (11) | 0.113 |
| 6 MWD achieved from predicted, %,median [IQR] (n) | 58 [34 – 78] (38) | 45 [31 – 61] (19) | 35 [26 – 55] (11) | 0.121 |
| Chair Stand Test, sec, median [IQR] | 10 [8-13] (38) | 12 [10-15] (10) | 12 [10-15] (10) | 0.528 |
| 4-m gait speed, m/sec, mean ± SD (n) | 0.91 ± 0.3 (38) | 0.73 ± 0.21 (16) | 0.68 ± 0.25 (13) | 0.031 |
| SPPB, 0-12, median [IQR] (n) | 11 [7 – 12] (38) | 9 [7 -10] (16) | 7 [4 – 11] (13) | 0.042 |
| MRC-ss, 0-60, median [IQR] (n) | 58 [54 – 60] (38) | 55 [51- 58] (16) | 52 [46 – 58] (13) | 0.025 |
| **HRQOL assessment** | | | | |
| EQ-5D VAS, 0-100, mean ± SD (n) | 77.9 ± 14.2 (54) | 68.9 ± 21.3 (21) | 69.7 ± 21.0 (17) | 0.066 |
| Return to work/hobby, yes, % (n) | 50% (29/58) | 28% (7/25) | 15% (3/20) | 0.009 |
| Return to driving, yes, % (n) | 70% (39/56) | 50% (11/22) | 42% (8/19) | 0.048 |
| **Cognitive function assessment** | | | | |
| MOCA, 0-30, median (IQR) | 24.8 ± 3.7 (48) | 24.4 ± 4.4 (20) | 25 ± 3.0 (17) | 0.979 |
| **Emotional health assessment** | | | | |
| Anxiety (HADS), 0-21, median [IQR] | 6 [2 – 11] (52) | 10 [5-13] (22) | 5 [1-8] (19) | 0.192 |
| Depression (HADS), 0-21, mean ± SD (n) | 5.3 ± 4.0 (52) | 7.3 ± 5.6 (22) | 6.5 ± 5.9 (19) | 0.418 |
| IESR-S, 0-88, mean ± SD (n) | 24.7 ± 21.6 (52) | 29.7 ± 21 (22) | 28 ± 27.6 (19) | 0.587 |
| **Clinical assessment** | | | | |
| 90-day hospitalization/ED visit, % (n) | 19% (11/59) | 28% (7/25) | 30% (6/20) | 0.493 |

**Supplemental Table 2**: Study outcomes of survivors of critical illness at 3 months following hospital discharge according to AKI and RRT status during index ICU admission

HADS = hospital anxiety and depression scale; IES-R = Impact of Events Scale-Revised; MOCA = Montreal Cognitive Assessment; ED =emergency department; EQ-5D = Euro-Quality of Life- Five-Dimension; VAS = visual analog scale; SPPB = short physical performance battery; MRC-ss = Medical research council-sum scale; 6 MWD = six-minute walk distance
